# Regulation of Sirtuin-3 and ERK1/2/p38MAPK by the combination Ga nanoparticles/γ-radiation low dosage: an effective approach for treatment of hepatocellular carcinoma

**DOI:** 10.1186/s43141-022-00385-6

**Published:** 2022-07-01

**Authors:** Mohga S. Abdalla, El-Sayed M. El-Mahdy, Somaya Z. Mansour, Sawsan M. Elsonbaty, Menna H. Amin

**Affiliations:** 1 Department of Chemistry, Faculty of Science, Helwan University, Helwan, Egypt; 2 Department of Radiobiology, National Center for Radiation Research and Technology, Egyptian Atomic Energy Authority, Nasr City, Egypt; 3 Department of Radiation Microbiology, National Center for Radiation Research and Technology, Egyptian Atomic Energy Authority, Nasr City, Egypt

**Keywords:** Hepatocellular carcinoma, Low dose γ-radiation, GaNPs, ERK1/2, p38MAPK, JNK_1_, Sirt3

## Abstract

**Background:**

Synthesized gallium nanoparticles synthesized by grape seed extract were characterized with spherical shape and size range less than100 nm, possessing the functional groups of the biological material. The purpose of this study is to evaluate gallium nanoparticles synthesized by grape seed extract, as an antitumor agent with low dose of γ-radiation against hepatocellular carcinoma in rats.

**Aim of work:**

This work aimed to evaluate the antitumor effect of gallium nanoparticles synthesized (GaNPs) by grape seed extract and the co-binded treatment with low dose of γ-radiation on hepatocellular carcinoma in rats, through evaluating their effect on signaling pathways and tumor markers.

**Results:**

Cytotoxic activity of GaNPs synthesized by grape seed extract was estimated by mediated cytotoxicity assay on HepG2 cell line that recorded IC_50_ of 388.8 μg/ml. To achieve these goals, eighty Wistar male rats (120−150 g) will be divided into eight groups, each of 10 rats. The animals are administered with diethylnitrosamine to induce hepatocellular carcinoma and then orally administered with GaNPs synthesized by grape seed extract (38.5 mg/kg) in combination with the exposure of the total body to a low dose of γ-radiation (0.5 Gy). The treatment modulated plasma vascular endothelial growth factor and alpha-fetoprotein. In addition, the immunoblotting results of nuclear factor-kappa beta showed a marked downregulation of extracellular signal-regulated kinase, mitogen-activated protein kinase, and c-Jun NH2-terminal kinase alongside, significantly elevating the level of Sirtuin-3 and caspase-3.

**Conclusions:**

It can be concluded that the combined treatment with GaNPs synthesized by grape seed extract and low dose γ-radiation may have antineoplastic activity against hepatocarcinogenesis by inhibiting signal pathways extracellular signal-regulated kinase/mitogen-activated protein kinase/c-Jun NH2-terminal kinase and stimulating apoptotic protein.

## Background

Hepatocellular carcinoma (HCC) is the world’s third most common cancer-related death [1]. The main causes of HCC include chronic hepatitis B and C infections [2]. Other factors that contribute to the formation of HCC include fatty liver disease, iron overload, alcoholism, and the exposure to environmental carcinogens [3]. Diethylnitrosamine (DEN) is one of the most prevalent carcinogens and frequently used for HCC induction. Simultaneously, it is widely used in the surroundings of everyday life, in tobacco, smoke, processed food, gasoline, and cosmetics [4].

Inorganic nanoparticles (NPs) have recently received more attention as potential cancer-fighting diagnostic and therapeutic systems. Recent studies have shown promising results in both in vitro and in vivo imaging and tumor therapy, as well. Definite metal nanoparticles and transition metals have been shown to have anticancer properties. Ga is the second most commonly used metal ion for cancer treatment, after platinum. Ga has several radionuclides that have been used in medicine to treat and diagnose illnesses. In addition to protein and DNA synthesis, and DNA inhibition, the activity of enzymes such as serum alkaline phosphatase is inhibited [5, 6]. Extensive exposure to Ga concentrations is expected to improve the therapeutic index of cancer diseases. The combination of biomolecules on the surface of the nanocomposite, such as phenols and flavonoids, alleviates cytotoxicity concerns, agglutination, and biological atmospheric instability and prevents cytotoxicity and the trapping of reactive oxygen species (ROS). In addition, Ga in the form of nanoparticles overcomes Ga tolerance in cancer therapy [7].

Grape seed extract (GSE) has recently received a lot of attention. Grapes (*Vitis vinifera*) are highly rich in polyphenols, as the seeds containing 60–70% of grape polyphenols, which can be used as a nutraceutical agent. Polyphenols, e.g., flavonoids and their polymers (proanthocyanidins), are abundant in grape seed extract, making it an excellent source of antioxidants [8, 9]. Because of its high polyphenols content and their structural variation, GSE has been shown to have cardioprotective, hepatoprotective, antidiabetic, antimutagenic, and anti-inflammatory effects [8, 10]. In addition, it has demonstrated promising chemopreventive and anticancer effects in a variety of cancer cells and animal tumor models, including skin, colorectal, prostate, and breast cancers [11, 12]. So far, only sporadic efforts have been made to investigate, mainly in vivo, the effect of GSE on liver cancer [13, 14].

Due to their intrinsic antitumor properties, metal nanoparticles may help to prevent tumor formation, development, and progression. The application of external stimuli has an extrinsic effect, such as in hyperthermia, where metal NPs are activated by external radiation such as IR or X-rays to form free radicals that destroy cancer cells and also enhance the cytotoxic impact of ionizing radiation [15, 16]. Ga is known to be the next most potent anticancer metal after platinum, as Ga NPs made in environmentally safe ways have shown anticancer efficacy against Ehrlich solid tumors via a redox mechanism [17].

Radiation hormesis is commonly assumed to mean that low-dose radiation in the region of 0.1–0.5 Gy has some physiologic advantage. The low dosage of radiation has been seen to activate the radical detoxification system and improve DNA repair rates. Furthermore, it has to raise immunological competence, which promotes the increase of a wide range of cytotoxic cells (lymphocytes), resulting in a decrease in the occurrence of metastatic cancer [18].

GaNPs combined with a low-dose of γ-radiation (RAD) have previously been shown to prevent the production of cytotoxic effects on cancer cells [17, 19]. Taking into account the abovementioned evidence on the potential of GaNPs as an anticancer agent, the present study was suggested to explore its potential therapeutic effect separately or combined with a low dose of γ-radiation against chemically induced hepatocellular carcinogenesis.

## Methods

### Materials

GSE will be obtained from pharmacological source (gravital capsules). DEN and Ga-nitrite were purchased from the Sigma-Aldrich Chemical Corporation, USA. Gallium nitrite (Sigma-Aldrich Company, Sigma-Aldrich, USA) dissolved in deionized water was used in the preparation of Ga NPs.

### γ-Irradiation

Whole-body γ-irradiation of rats was performed using the Canadian gamma cell-40 (137Cs) at the National Center for Radiation Research and Technology (NCRRT), Cairo, Egypt. Rats were exposed to a single dose of γ-radiation (0.5 Gy) at a dose rate 0.912 rad/s. Dosimetry was performed using a 5-mm diameter alanine dosimeter (Bruker Instruments, Rheinstetten, Germany), and the free radical signal was measured using an ESR analyzer (EMXplus, Bruker Instruments, Rheinstetten, Germany) to evaluate the actual doses.

#### Experimental animals

Wistar male rats weighing 120–150 g were obtained from the Nile Pharmaceutical and Industries Co. at Amiria, Cairo. Rats were housed in plastic cages, five rats in each cage freely fed of commercial diet (21% protein), excess of drinking water, and temperature range 22 ± 3 °C at the animal house of the NCRRT. They were allowed to acclimatize to the environmental conditions such as temperature, pressure, humidity, good ventilation, and illumination conditions for 1 week before the experiment. All rats were cared in accordance with the ethics committee of the NCRRT according to the “guide for the care and use of laboratory animals” published by the US National Institute of Health [20].

### Preparation of GaNPs

First, 8.0 g of grape seed powder was mixed with 100 ml of distilled water, and the mixture resulted was placed in a water bath for 30 min at a temperature of 60 °C. Next, the solution was filtered with Whatman filter paper no. 1, and then, the filtrate (extract) was stored at 4 °C. GaNPs synthesis was performed, according to the method described by Mohsen et al. [21]. At the initial stage, a freshly prepared gallium nitrite solution (1 mM, alkaline pH) was added to the seed extract solution at the ratio of 1:4.

### Characterization of GaNPs

#### Transmission electron microscopy

Synthesized GaNPs size and shape were analyzed by transmission electron microscope (TEM). TEM sample was prepared by placing a drop of GaNPs suspension on carbon-coated copper grids and allowing water to evaporate. The size of nanoparticles was determined from TEM micrographs performed by JEOL model 1200EX. The software (advanced microscopy techniques, Danvers, MA, USA) for the digital TEM camera was calibrated for nanoparticles size measurement.

#### UV-spectrophotometric analysis

Reduction of Ga ions in GaNPs was monitored by UV-visible spectroscopy (Jenway UV spectrophotometer model 6505) at the wavelength range of 200–600 nm. An amount of 0.2 ml of GaNPs aliquots was diluted with 1.8 ml of distilled water, and then, the absorbance was recorded. Liquid and powder forms of the sample were used for further characterization.

#### Dynamic light scattering (DLS)

Sample of GaNPs was analyzed for size dimensions by DLS Zetasizer (ZS, Malvern, UK).

#### In vitro cytotoxicity of Ga NPs on HepG2 cell line

It was estimated by the measurement of the IC_50_ values for Ga NPs samples against the HepG2 cell line using sulforhodamine B assay for cytotoxicity [22].

#### Cell line and cell culture

Human hepatocellular carcinoma (HepG2) cell line was obtained from the Egyptian National Cancer Institute, Cairo University. HepG2 cell lines were cultured using RPMI 1640 media, with 10% FBS, 1% P/S, and 1% L-glutamine obtained from Life Technologies, Gibco (Grand Island, NY). Cells were cultured in 5% CO_2_ at 37 °C and then treated with 0.25% (w/v) trypsin/EDTA to affect cell release from the culture flask.

#### Cell viability assay

Cell viability refers to the number of live, healthy cells in a sample [23]. Cell viability assays are used to measure the physical and physiological health of cells in response to extracellular stimuli, chemical agents, or therapeutic treatments [23, 24]. Cell viability is the ratio of initial cell number minus dead cell number to the initial cell number. Briefly, cells were cultured in a 96-well plate for 24 h, and fresh medium containing various concentrations of GaNPs (0–50 μg/ml) was added and incubated for 24 h. HepG2 cells were fixed with ice-cold 10% trichloroacetic acid at 4 °C; stained with 0.4% sulforhodamine B (SRB), a fluorescent dye to the quantification of cellular proteins of cultured cells, for 30 min at room temperature; and dissolved with 10 mM Tris base solution. Soluble dye absorbance was measured spectrophotometrically at 510 nm.

### In vivo study

#### Determination of LD_50_

Determination of LD_50_ was performed on male Wistar rats experimental animals. The LD_50_ of newly synthesized GaNPs was determined in as preliminary step to in vivo study according to Akhila et al. [25]. According to recommended methodology of Akhila et al. [25], thirty (30) male Wistar rats were used for this study; thirty rats were separated into six groups of 5 rats each. The five groups of rats (GRP 1–6) were administered GaNPs orally at concentrations 10, 30, 50, 100, 150, and 300 mg/kg body weight, respectively, and observed for signs of toxicity like behavioral changes, increased respiratory rate, nervous imbalance, and death within 48 h there were no signs of toxicity or mortality.

#### Experimental design

In the present study, 80 male Wistar rats were divided into eight groups, each of 10 rats.

- Group 1 (control, C): Animals received 1 ml of physiological saline orally by gavage.
- Group 2 (RAD): Rats whole body was exposed once to low dose of γ-radiation (0.5 Gy).
- Group 3 (GaNPs): Rats received GaNPs (10% of LD_50_ dose 38.5 mg/kg) orally by gavage.
- Group 4 (GaNPs + RAD): Rats received GaNPs, five times a week for 6 weeks, and exposed to 0.5 Gy γ-radiation dose.
- Group 5 (DEN): Each animal received DEN (dissolved in 0.9% normal saline), orally by gavage (20 mg/kg, 5 times/week for 6 weeks).
- Group 6 (DEN + GaNPs): Rats received DEN as group 5 and then treated with GaNPs for 6 weeks as in group 3.
- Group 7 (DEN + RAD): Rats received DEN as in group 5 and then exposed to 0.5 Gy γ-radiation.
- Group 8 (DEN + GaNPs + RAD ): Rats received DEN as in group 5, then treated with GaNPs for 6 weeks as in group 3, and finally exposed to 0.5 Gy γ-radiation.

#### Sample processing

Twenty-four hours after the last treatment, all animals were anesthetized with urethane (1.2 g/kg BW, Sigma-Aldrich, St. Louis), [26]. Blood samples were collected from rats under light ether anesthesia using capillary tube. The collected blood in EDTA tubes was centrifuged, and separated serum used in determining some parameters and liver was immediately isolated, washed by ice-cold physiological saline, dried, and preserved for subsequent analysis at −80 °C. Tissue samples of the livers were fixed in a 10% neutral buffered formalin solution for histopathological investigation. In the ascending ethanol concentration, tissue specimens were dehydrated, cleared in xylene, implanted in paraffin wax and sectioned at a thickness of 5 μm, and stained by hematoxylin and eosin (H&E) [27].

#### Biochemical assay

ELISA kits for rats (MyBioSource, USA) were used for quantifying of serum: alpha-fetoprotein (AFP, catalog no: MBS700622), vascular endothelial growth factor (VEGF, catalog no: MBS9501942), nuclear factor-kappa beta (NF-κb, catalog no: MBS722386), and caspase-3 (Casp-3, catalog no: MBS018987) levels in the liver homogenate. The analysis was performed according to the manufacturer’s protocol for the commercial kits. For the preparation of liver tissue homogenate, 1.0 g of the liver tissue was homogenized in 10.0 ml of phosphate-buffered saline (PBS) using glass homogenizer on ice.

#### Western blot analysis

To investigate the changes in mRNA expression for ERK1/2, MAPK38 and JNK1, and β-actin genes, total RNA was isolated from liver tissue using TRIzol reagent (Invitrogen) to extract liver tissue proteins. Protein concentration was estimated using Protein Assay kit (Bio-Rad Laboratories, Hercules, CA, USA). The desired protein band was isolated by 10% sodium dodecyl sulfate polyacrylamide gel electrophoresis (SDS-PAGE). Equal amounts of protein extracts (50 mg) were loaded per lane of SDS-PAGE. Protein bands were transferred from SDS-PAGE into polyvinylidene fluoride or polyvinylidene difluoride (PVDF) membrane. The membrane was blocked with 5% nonfat milk for 2 h; incubated with the primary rat polyclonal antibodies (Invitrogen, Thermo Fisher, USA), ERK1/ERK2 Polyclonal Antibody (cat no. 61-7400), p38 MAPK beta Polyclonal Antibody (Cat no. PA1-41154), and β-actin as control gene (catalog no: PA5-85490); and then incubated together with the secondary monoclonal antibody. Then, it was conjugated with horseradish peroxidase (Invitrogen, Thermo Fisher, USA, Catalog no: PA1-29927), at room temperature, for 2 h. Quantification of ERK_1/2_, p38MAPK, JNK_1_, and β-actin proteins was carried out using scanning laser densitometer analysis (Biomed Instrument Inc., USA). The level of expression was normalized to β-actin protein level. Proteins level was estimated by densitometry analysis, using Bio-Rad software, USA (Clarity™ Western ECL substrate — Bio-Rad, USA cat no. 170-5060). Densitometry data generated for Western blots are commonly used to compare protein abundance between samples. Nonlinear densitometry data were observed when Western blots were detected using infrared fluorescence or chemiluminescence and under different SDS-PAGE conditions

#### Quantitative real-time PCR of Sirt-3

Quantitative Sirt-3 gene expression was evaluated. Total RNA was isolated from the liver tissue homogenate by using TRIzol (Invitrogen) according to the protocol of the manufacturers. Reverse transcription of the extracted mRNA samples was performed by transcriptase enzyme. A SuperScript kit from Invitrogen was used to prepare cDNA. RT-PCRs were performed in a thermal cycler StepOnePlus™. The oligonucleotides primer gene of NAD-dependent deacetylase Sirtuin-3 is as follows: F-5′ AAGACATACGGGTGGAGCCT, R-5′ GGACTCAGAGCAAAGGACCC, and for β-actin: F-5′ CCCGCGAGTACAACCTTCTT, R-5′ CGACGAGCGCAGCGATA. PCR thermal-cycling conditions included an initial step at 95 °C for 5 min, 40 cycles at 95 °C for 20 s, 60 °C for 30 s, and 72 °C for 20 s. Relative expression of Sirt-3mRNA was calculated according to Pfaffl’s calculations [28] that performed by normalizing the average Ct value of each treatment compared to the endogenous control gene β-actin.

### Calculation of relative quantification (RQ) (relative expression)

The relative quantitation was calculated according to Applied Biosystem software using the following equation:

$$\varDelta \mathrm{Ct}=\mathrm{Ct}\ \mathrm{gene}\ \mathrm{test}-\mathrm{Ct}\ \mathrm{endogenous}\ \mathrm{control}$$

$$\varDelta \varDelta \mathrm{Ct}=\varDelta \mathrm{Ct}\ \mathrm{sample}1-\varDelta \mathrm{Ct}\ \mathrm{calibrator}$$

$$\mathrm{RQ}=\mathrm{Relative}\ \mathrm{quantification}={2}^{-\varDelta \varDelta \mathrm{Ct}}$$

The RQ is the fold change compared to the calibrator (untreated sample).

### Statistical analysis

Statistical analysis of the results was performed using the statistical package for Windows Version 15.0 (SPSS Software, Chicago, IL). The results for continuous variables were expressed as a mean ± standard error. Values were compared by one-way analysis of variance (ANOVA). Post hoc testing was performed for intergroup comparisons using the least significant difference test, and significance of *p* values, *p* ≥ 0.05, was considered statistically significant.

## Results

### Characterization of Ga NPs

**Fig. 1:**
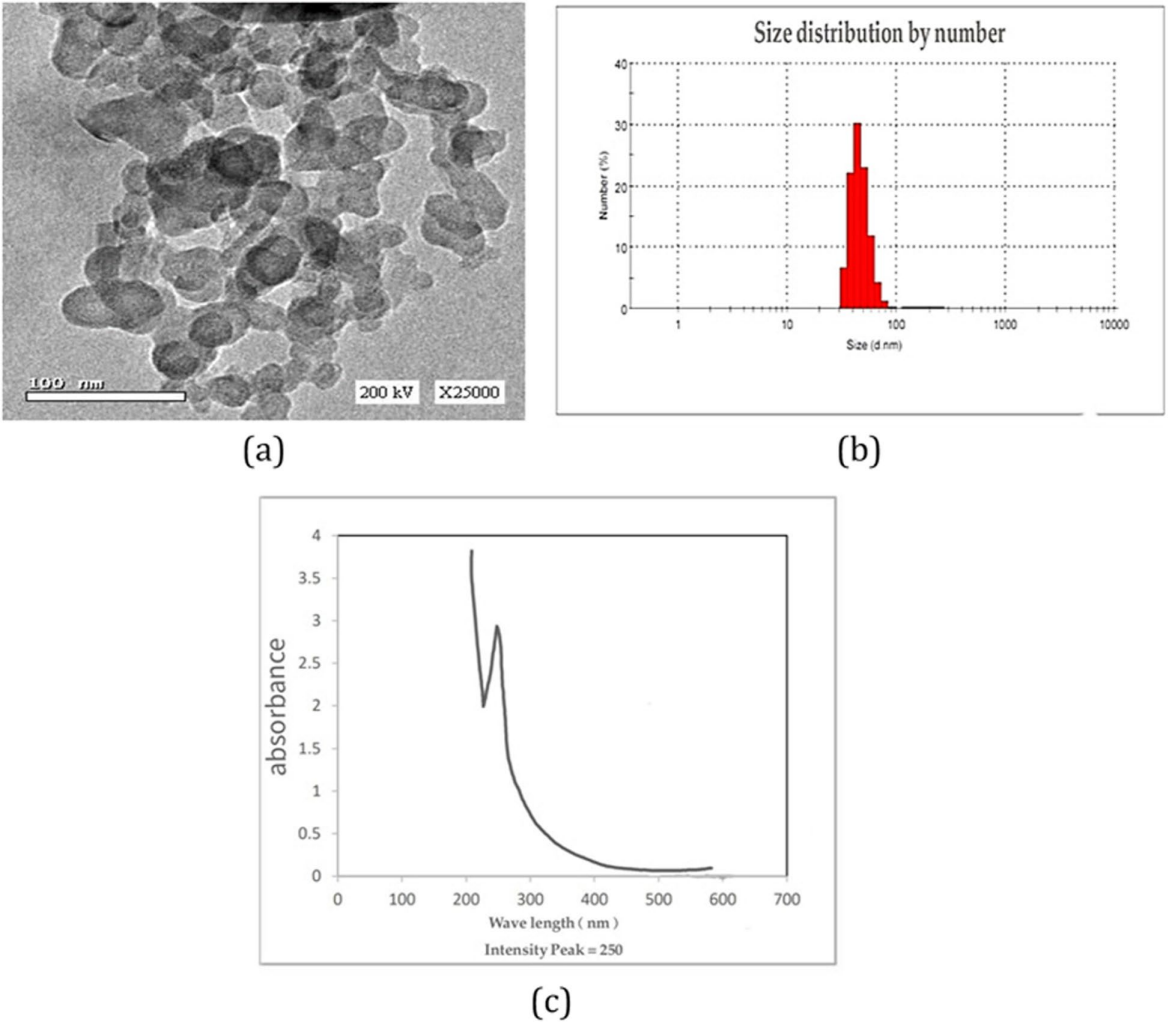
**a** Transmission electron microscope photo. **b** DLS analysis of GaNPs for size determination. **c** UV-Vis absorbance of GaNPs synthesized by grape seed extract, a sharp peak of absorbance at 250 nm

The size and morphology of the biosynthesized GaNPs were portrayed by means of TEM photograph investigation. TEM pictures of the GaNPs affirmed their round shape with a generally restricted molecule size. The depicted ball-like structure of the GaNPs in the TEM images recorded a size range of less than 100 nm in diameter (Fig. 1a), confirming the presence of a layer covering round the nanoballs.

DLS estimated the size of the biosynthesized GaNPs to be in the range of 32.67–255.2 nm measuring the size of nanoparticles with the hydrodynamic layer around it. Size distribution was classified into high distribution for nanoparticles of 37.84, 43.82, and 50.77 nm with distribution percentage of 24.7, 32.5, and 8.3%, respectively. Polydispersity index (PDI) was of 0.795, demonstrating the homogeneity and uniform dispersing of the incorporated GaNPs (Fig. 1b). The development of GaNPs was affirmed by UV-spectroscopy that results in a sharp peak of absorbance at 250 nm relegated to the surface plasmon reverberation for the shaped nanoparticles.

### In vitro cytotoxicity of GaNPs on HepG2 cell line

**Fig. 2:**
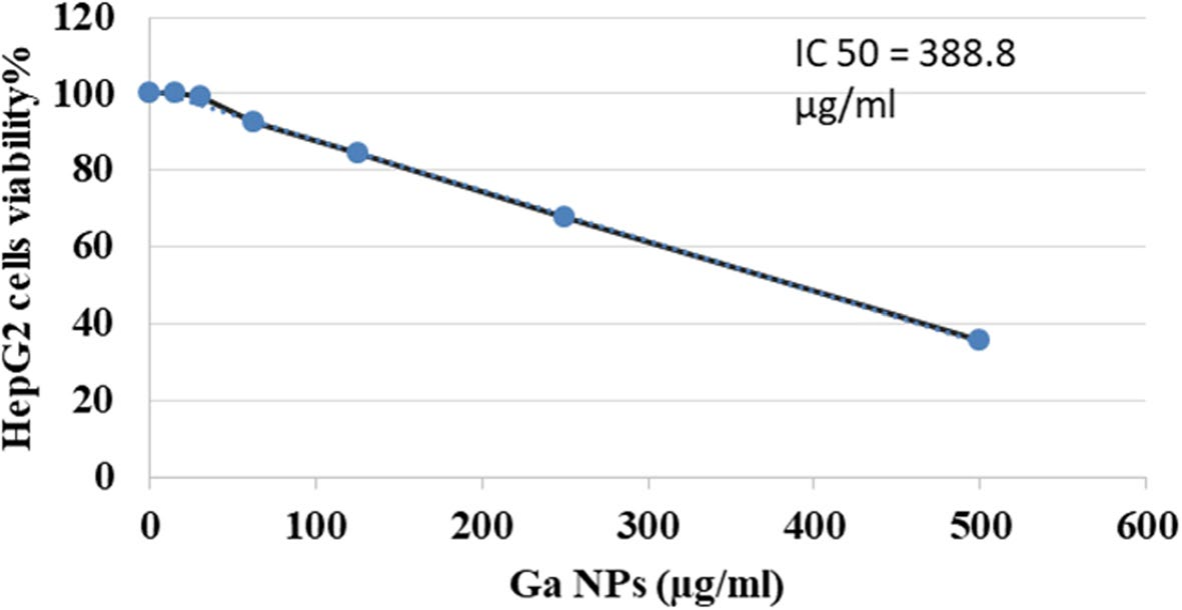
Cytotoxicity effect of GaNPs on HepG2 cell line

The cytotoxic activity of Ga NPs was assessed utilizing HepG2 cell line. The synthesized GaNPs was applied at various concentrations and clearly exhibited a cytotoxic effect against HepG2, at all of the examined concentrations (100, 200, 330, 400, and 500 μg/ml). In a concentration-dependent manner of GaNPs, cytotoxic activity increased gradually up to 64.16% at the concentration of 500 μg/ml, with IC_50_ value of 388.8 μg/ml (Fig. 2).

### In vivo studies

#### Determination of ERK1_/2_, p38 MAPK, and JNK_1_levels

**Fig. 3:**
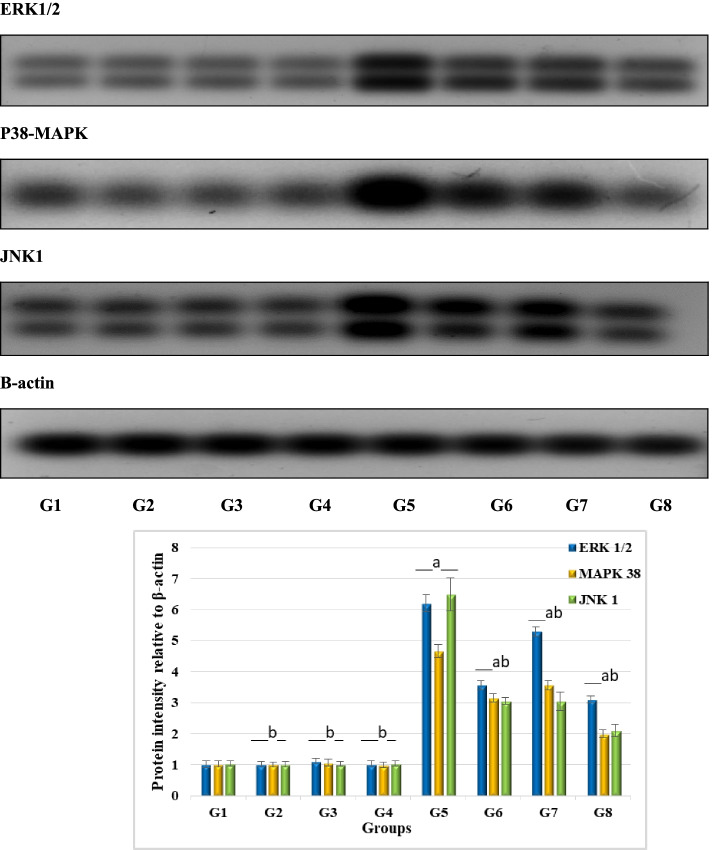
Western immunoblotting analysis of signaling growth factors: ERK1/2, p38-MAPK, and JNK1 protein expression in treated rats groups. G1 control, G2 RAD, G3 GaNPs, G4 GaNPs + RAD, G5 DEN, G6 DEN + GaNPs, G7 DEN + RAD, G8 DEN + GaNPs + RAD. Each bar represents mean ± SD. ^a^*P* < 0.05 compared to control; ^b^*P* < 0.05 compared to DEN group

The ERK_1/2_, p38 MAPK, and JNK_1_protein expression ratio to β-actin was assessed in the liver tissues of different experimental groups by Western blot analysis. Administration of DEN to healthy rats significantly elevated liver protein intensity of ERK1/2, p38MAPK, and JNK1; they found to be 6.20 ± 0.27, 4.67 ± 0.21, and 6.50 ± 0.53, respectively, while the control group recorded 1.02 ± 0.10, 1.03 ± 0.09, and 1.04 ± 0.09 for the same signaling growth factors. Injecting DEN-intoxicated rats with GaNPs or exposure to radiation significantly ameliorated ERK_1/2_, p38 MAPK, and JNK_1_ protein expression; they get better to reach 3.56 ± 0.15, 3.15 ± 0.14, and 3.05 ± 0.10 for group 6 (DEN + GaNPs) and 5.30 ± 0.15, 3.56 ± 0.15, and 3.04 ± 0.30 for group 7 (DEN + RAD) in regard to group 5 (DEN-treated rats). Concerning to group 8 (DEN + GaNPs + γ-RAD), the combined treatment demonstrated the best effect in the expression levels of ERK1/2, p38MAPK, and JNK1 to become 3.10 ± 0.12, 1.99 ± 0.12, and 2.10 ± 0.20, respectively, versus DEN group (Table 1; Fig. 3).

**Table 1:** Effect of different treatments on Western immunoblotting analysis of signaling growth factors: ERK1/2, p38-MAPK, and JNK1 protein expression in DEN-treated rats (^a^*P* < 0.05 compared to control; ^b^*P* < 0.05 compared to DEN group)

Parameter	G1 Control	G2 RAD	G3 GaNPs	G4 GaNPs + R	G5 DEN	G6 DEN + GaNPs	G7 DEN + RAD	G8 DEN + GaNPs + RAD
ERK1/2	1.02 ± 0.10	1.02 ± 0.09b	1.12 ± 0.08b	1.03 ± 0.10b	6.20 ± 0.27a	3.56 ± 0.15ab	5.30 ± 0.15ab	3.10 ± 0.12ab
p38-MAPK	1.03 ± 0.09	1.01 ± 0.08b	1.07 ± 0.10b	0.99 ± 0.08b	4.67 ± 0.21a	3.15 ± 0.14ab	3.56 ± 0.15ab	1.99 ± 0.12ab
JNK1	1.04 ± 0.09	1.02 ± 0.09b	1.03 ± 0.08b	1.04 ± 0.09b	6.50 ± 0.53a	3.05 ± 0.10ab	3.04 ± 0.30ab	2.10 ± 0.20ab

#### Impact on SirT-3 gene expression

**Fig. 4:**
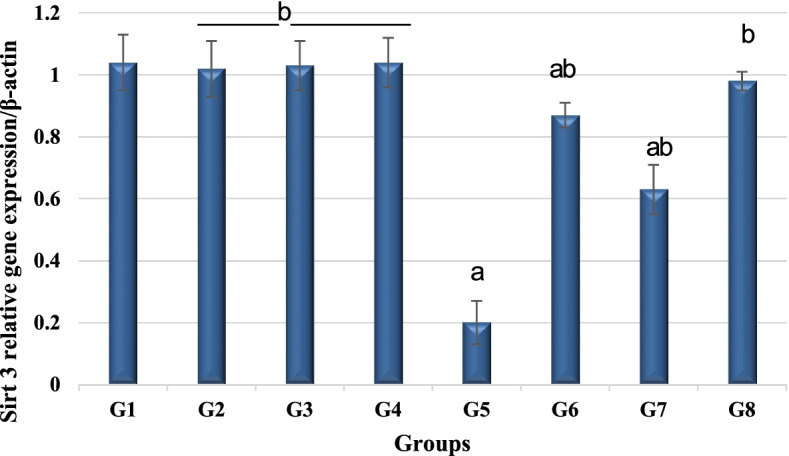
Quantitative gene expression of liver SirT-3 in different groups. G1 control, G2 RAD, G3 GaNPs, G4 GaNPs + RAD, G5 DEN, G6 DEN + GaNPs, G7 DEN + RAD, G8 DEN + GaNPs + RAD. Each bar represents mean ± SD. ^a^*P* < 0.05 compared to control; ^b^*P* < 0.05 compared to DEN group

In contrast to the control healthy rats, the results of qrt PCR emphasized that liver SirT-3 mRNA level of rats induced to promote HCC by DEN treatment was significantly downregulated. SirT-3 gene expression was significantly upregulated in group 6 (DEN + GaNPs), group 7 (DEN + RAD), and combined treatment of GaNPs and γ-RAD (group 8); SirT-3 gene found to be 0.87 ± 0.04, 0.63 ± 0.08, and 0.98 ± 0.03, respectively, in relation to group 5 (DEN), which recorded 0.20 ± 0.07. The results clearly showed normalization of SirT-3 mRNA level and mitigate DEN inhibitory effect in comparison with DEN rats due to the treatments applied (Table 2; Fig. 4).

**Table 2:** Effect of different treatments on quantitative gene expression of liver SirT-3 in DEN-treated rats (^a^*P* < 0.05 compared to control; ^b^*P* < 0.05 compared to DEN group)

Parameter	G1 Control	G2 RAD	G3 GaNPs	G4 GaNPs + R	G5 DEN	G6 DEN + GaNPs	G7 DEN + RAD	G8 DEN + GaNPs + RAD
SirT-3	1.04 ± 0.09	1.02 ± 0.09b	1.03 ± 0.08b	1.04 ± 0.08b	0.20 ± 0.07a	0.87 ± 0.04ab	0.63 ± 0.08ab	0.98 ± 0.03b

#### Determination of VEGF, NF-κb, and AFP levels

**Fig. 5:**
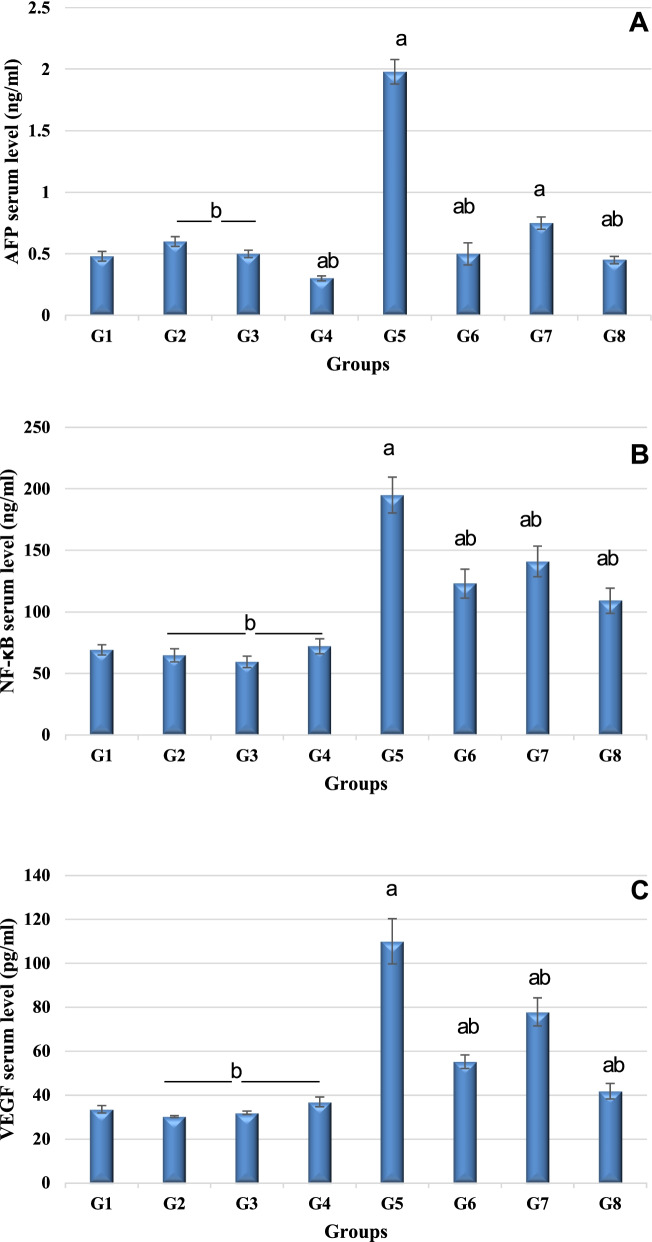
**A** Serum level of AFP. **B** Serum level of NF-kB. **C** Serum level of VEGF of different treated groups. G1 control, G2 RAD, G3 GaNPs, G4 GaNPs + RAD, G5 DEN, G6 DEN + GaNPs, G7 DEN + RAD, G8 DEN + GaNPs + RAD. Each bar represents mean ± SD. ^a^*P* < 0.05 compared to control; ^b^*P* < 0.05 compared to DEN group

As an indication for the carcinogenic state of the liver, it was observed that the serum level of VEGF, NF-κb, and AFP, analyzed using ELIZA, was significantly elevated in DEN-treated rats (group 5). Meanwhile, serum level of VEGF, NF-κb, and AFP was significantly reduced in rats subjected to DEN administration and treated with GaNPs, γ-RAD, and GaNPs + γ-RAD (groups 6, 7, and 8). Serum levels of VEGF, NF-κb, and AFP decreased to be 0.50 ± 0.09, 123.0 ± 11.8 μg/ml, and 55.1 ± 3.09 pg/ml for group 6 and 0.75 ± 0.05, 141.0 ± 12.5 μg/ml, and 77.8 ± 6.4 pg/ml for group 7, while group 8 recorded 0.48 ± 0.04 and 69.1±4.2 μg/ml for VEGF and NF-κb alongside 33.5 ± 1.7 pg/ml for AFP, which was somewhat closer to the non-treated rats of group 1 (Table 3; Fig. 5).

**Table 3:** Effect of different treatments on VEGF, NF-kB, and AFP in DEN-treated rats (^a^*P* < 0.05 compared to control; ^b^*P* < 0.05 compared to DEN group)

Parameter	G1 Control	G2 RAD	G3 GaNPs	G4 GaNPs + R	G5 DEN	G6 DEN + GaNPs	G7 DEN + RAD	G8 DEN + GaNPs + RAD
**AFP** **(ng/ml)**	0.48 ± 0.04	0.6 ± 0.04b	0.5 ± 0.03b	0.3 ± 0.02ab	1.98 ± 0.1a	0.50 ± 0.09ab	0.75 ± 0.05ab	0.45 ± 0.03ab
**NF-κB** **(ng/ml)**	69.1 ± 4.2	64.7 ± 5.3b	59.3 ± 4.7b	72.0 ± 6.1b	195.0 ± 14.6a	123.0 ± 11.8ab	141.0 ± 12.5ab	109.0± 10.3ab
**VEGF** **(pg/ml)**	33.5 ± 1.7	30.2 ± 0.46b	31.9 ± 0.75b	36.8 ± 2.22b	110 ± 10.3a	55.1 ± 3.09ab	77.8 ± 6.4ab	41.7 ± 3.5ab

#### Determination of Caspase-3 level

**Fig. 6:**
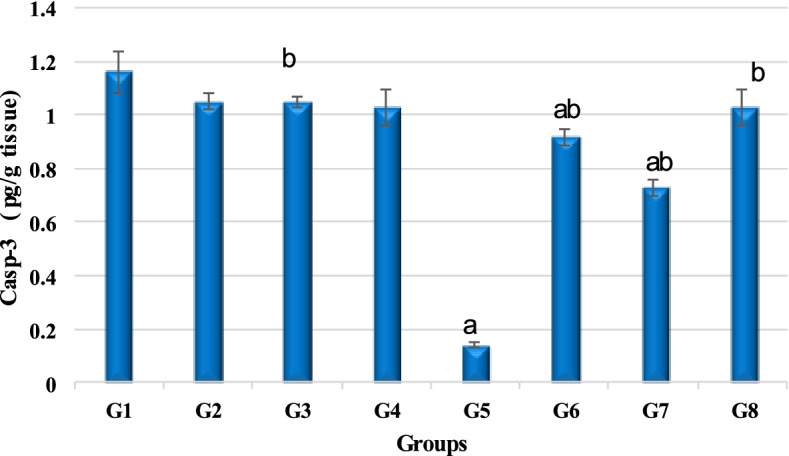
Caspase-3 level affected by treatments in different groups. G1 control, G2 RAD, G3 GaNPs, G4 GaNPs + RAD, G5 DEN, G6 DEN + GaNPs, G7 DEN + RAD, G8 DEN + GaNPs + RAD. Each bar represents mean ± SD. ^a^*P* < 0.05 compared to control; ^b^*P* < 0.05 compared to DEN group

Toward determination the effect of different treatments on liver cells apoptosis, caspase-3 concentration was analyzed. Results of the caspase-3 level analysis exhibited a significant reduction in the liver tissue of the DEN-intoxicated rats (0.14 ± 0.02 pg/g tissue) in contrast to normal healthy rats of group that revealed 1.16 ± 0.10 pg/g tissue. Moreover, results revealed that rats intoxicated with DEN and treated with GaNPs (group 6), γ-RAD (group7), and GaNPs + γ-RAD (group 8) showed significant elevation in Caspase-3 concentration, i.e., 0.92 ± 0.03, 0.73 ± 0.06, and 1.03 ± 0.07 pg/g tissue that can be considered to be very close to the value of control group (1.16 ± 0.10 pg/g tissue (Tables 4; Fig. 6).

**Table 4:** Effect of different treatments on Caspase-3 in DEN-treated rats (^a^*P* < 0.05 compared to control; ^b^*P* < 0.05 compared to DEN group)

Parameter	G1 Control	G2 RAD	G3 GaNPs	G4 GaNPs + R	G5 DEN	G6 DEN + GaNPs	G7 DEN + RAD	G8 DEN + GaNPs + RAD
Caspase-3 (pg/g tissue)	1.16 ± 0.10	1.05 ± 0.08b	1.05 ± 0.08b	1.03 ± 0.09b	0.14 ± 0.02a	0.92 ± 0.03ab	0.73 ± 0.06ab	1.03 ± 0.07b

## Discussion

Based on the fact that the combination of therapies is a successful and more effective strategy for cancer treatment, chemotherapy is combined with radiotherapy, increasing the effects of cancer treatment and making cancer therapy more effective. Chemoradiotherapy can be improved both chemotherapy and radiotherapy effectiveness. In addition, guided treatments with NPs and radiotherapy could be an effective strategy in cancer therapy to overcome limitations in conventional chemotherapy. Because of their tiny size, NPs are able to permeabilize cells effectively, which facilitates activity and in vivo distribution. Because they are not captured by the reticuloendothelial system, smaller NPs accumulate more in the tumor regions and also have a prolonged in vivo half-life [29, 30]. Targeted NPs for HCC therapy are more effective than for other types of cancers as most of which might end up in the liver and spleen [31].

Previous research has shown that GabNPs produced by *Bacillus helveticus* bacteria have antiproliferative capabilities in MCF-7 [17] and HepG2 cell lines [30]. In vivo studies demonstrated antiproliferative and proapoptotic effects against Ehrlich solid tumors in mice and HCC in rats [17, 32]. This prompted us to do more research on GaNPs antitumor mechanisms as a viable alternative drug for cancer therapy and a radiosensitizing agent.

In this study, biosynthesized GaNPs using grape seed extract were characterized by various analysis methods (TEM, DLS, and UV/VIS spectroscopy). The findings revealed that the GaNPs have shown a diameter of less than 100 nm and a characteristic peak at 250 nm assigned to the surface plasmon resonance of the NPs and Ga sensitivity to UV radiation below the 365-nm wavelength. The functional groups of the reducing agent used in the synthesized GaNPs were approved by FTIR analysis. These facts were in good agreement with that biosynthesized by bacterial extracellular extract as it recorded a diameter of the range 8–20 nm and UV/VIS absorbance peak at 265 nm [17].

In vitro antiproliferative activity of GaNPs synthesized by grape seed extract showed less cytotoxicity (*IC*_50_ = 388 g/ml) than that synthesized by *Bacillus helveticus* (*IC*_50_ = 8.0 g/ml) against HepG2 [17]. This can be explained due to the reducing capability of the material used in the synthesis as a reducing and capping agent for Ga [33].

Along with reduced glutathione (GSH), imbalance between ROS formation as a result of DEN administration and antioxidant scavengers seriously damage biological systems and promote carcinogenesis by injuring tissues, causing chromosomal instability, altering biochemical compounds, and eroding cell membranes and mutation, which are involved in all stages of carcinogenesis, i.e., initiation, promotion, and progression [34].

The present work emphasizes that induction of HCC in male rats through DEN administration markedly suppressed Sirt-3 expression in the liver. Sirt-3 is a key control for many pathways of cancer cell and has a prognostic value in HCC patients [35]. Sirt-3 protein is demonstrated for NAD-dependent protein deacetylase activity suggesting a probable function as cellular sensors of metabolic or oxidative states and accordingly regulates cellular functions [36]. Sirt-3 is positively correlated with SOD2 and plays a role in its expression [35] and control ROS homeostasis in the cell. Sirt-3 level reduction derived the cell into chromosomal, genetic instability, and biochemical alterations as results for ROS level elevation. Consequently, the elevation of ROS level induces the expression of hypoxia-inducible factor 1α (HIF-1α), which stimulates nuclear expression of proliferative signal of VEGF [37]. VEGF plays a critical role during angiogenesis and tumor growth [38].

Treating DEN rats with GaNPs and low dose of γ-radiation significantly elevated Sirt-3 expression which may play a role in tumor suppression [39], mainly through mediating the suppression of hypoxia-inducible factor 1α (HIF-1α) and inhibiting mitochondrial ROS production [40, 41]. SIRT-3 is well known for its ability to eliminate reactive oxygen species and to prevent the development of cancerous cells or apoptosis [42]. The proliferation-suppressor role of Sirt-3 was confirmed in multiple cancer types, including breast cancer and colon cancer, both in vitro and in vivo [41]; it was also reported that Sirt-3 could inhibit HCC cell growth through reducing Mdm2-mediated p53 degradation [43].

Gallium is capable to inhibit tumor growth, mainly because of its competition to ferric and magnesium ions. Gallium affects cellular acquisition of iron by binding to transferrin besides its interaction with the iron-dependent enzyme ribonucleotide reductase and inhibition of DNA synthesis [44]. Gallium also provokes DNA fragmentation which stimulates cellular apoptosis [17]. Iron is important for cellular respiration. ROS produced by iron stimulate various signaling pathways, including mitogen-activated protein kinase (MAPK) signaling pathways, and include the apoptosis signal-regulating kinase 1 (ASK1)-p38/JNK pathway [45].

Gallium may interact with DNA through its competition with magnesium for binding to DNA as a result of Ga high affinity to DNA 100 times than magnesium [46]. Ga causes DNA structural modifications by binding to DNA phosphate group and nucleic bases. Previous investigations showed that gallium activates caspases and induces apoptosis through the mitochondrial pathway [47]. The present study results indicated the inhibitory effect of GaNPs on the growth of tumor cells and induction of apoptosis as caspase-3 was significantly increased with morphological changes typical of apoptosis. GANPs formulation is a potential candidate for the prevention and treatment of hepatic and breast cancers [48].

The current study shows a significant increase in the serum level of AFP in the DEN group, which agreed with that of Song et al [49]. The increase in AFP by neoplastic hepatocytes is either due to increased transcription of AFP gene or posttranslational modification. AFP production is roughly proportional to the amount of transplantable mRNA present in rats exposed to chemical carcinogens or induced HCC [50]. Administration of GaNPs and its combination with the low dose of γ-radiation significantly limited the elevation of serum AFP level as compared with the DEN intoxicated group, suggesting that GaNPs and low dose of radiation might delay the hepatocarcinogenesis and deactivate neoplastic cells production.

An important key parameter in cancer cell is the classic MAP kinase family consists of three subfamilies: extracellular signal-regulated kinase (ERK), c-Jun N-terminal kinase (JNK), and p38-MAP kinase. In this work, DEN markedly elevated p38-MAPK, ERK1/2, and JNK1 levels. They are signaling pathways responsive to a large number of extracellular stimuli and elevated in cancer cells in response to proliferation stimuli. ERK1/2 is activated to inhibit apoptosis in response to a wide range of stimuli, such as tumor necrosis factor (TNF), Fas ligand, TNF-related apoptosis-inducing ligand (TRAIL) [51], osmotic stress [52], and hypoxia [53]. Inhibition of p38 MAPK stimulates Raf and ERK activities and induces myoblast proliferation [54]. Activation of p38MAPK results in rapid dephosphorylation of MEK1/2 and subsequent apoptosis. In this work, GaNPs and low dose of γ-radiation ameliorated p38MAPK, ERK1/2, and JNK1 levels, which direct cellular mechanism against carcinogenesis and toward apoptosis. The inactivation of NF-κB and ERK/JNK/MAPK signaling pathways involve in controlling cancer cell proliferation.

Regarding the apoptotic marker caspase-3, its concentration was reduced by administration of DEN, which was induced by GaNPs and/or radiation treatment compared to non-treated rat. GaNPs and radiation may induce apoptosis by upregulation of caspase-3. Gallium in the form of gallium nitrate induces apoptosis primarily through the mitochondrial pathway in CCRF-CEM cells through activation of Bax, the release of cytochrome C, and the activation of caspase-3 [55]. It was reported that the exposure to low dose of γ-radiation suppressed neoplastic transformation from a high challenge dose by stimulating intra- and intercellular signaling, leading to activated natural protection (ANP) against genomic instability-associated diseases, such as cancer [56]. The activation ANP may include induced p53-dependent high-fidelity DNA repair along with normal apoptosis activation of an epigenetic-protective apoptosis-mediated process, which selectively removes premalignant transformed cells while also enhances immune functions [57].

Grape seed also plays a role in stimulating caspase-3. The activation of caspase-3 by grape seed either through apoptotic ligand- or mitochondria-mediated activation of the caspase cascade may be a potential mechanism underlying GS-induced apoptosis in leukemia cells. Also, activation of JNK significantly enhanced caspase activation and apoptosis [58]. Finally, cancer’s rising prominence as a leading cause of death partly reflects marked declines in mortality rates of stroke [59].

## Conclusions

From the obtained results, it can be concluded that the combined treatment with GaNPs and low-dose γ-radiation may have antineoplastic activity against hepatocarcinogenesis by inhibiting signal pathways p38MAPK/ERK1/2/JNK1 and stimulating apoptotic protein.

### Recommendation

It is recommended to use gallium nanoparticles (GaNPs) synthesized by grape seed with low dose of gamma radiation for early diagnosis and cure of hepatocarcinogenesis, which may enhance biochemical parameters against damaging effect of carcinogenic compound.

## Data Availability

The datasets used and/or analyzed during the current study are available from the corresponding author on reasonable request.
